# Supplementary figures and images for: Metabolic Predictors of Equine Performance in Endurance Racing
Source: Metabolites. 2021 Jan 31;11(2):82. doi: 10.3390/metabo11020082 (PMC7912089; doi:10.3390/metabo11020082)

FigS1

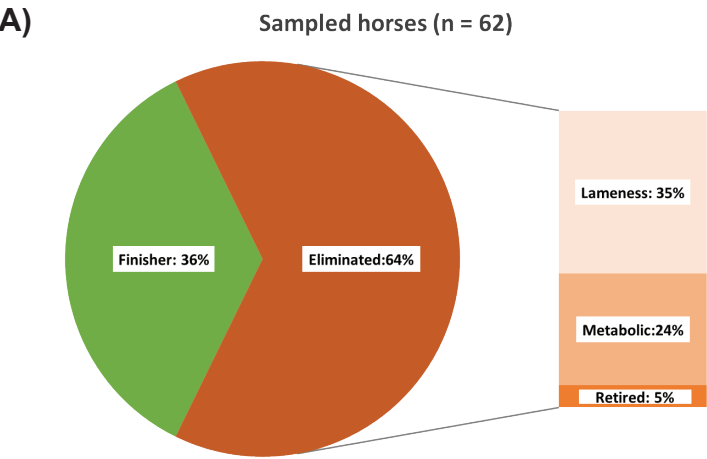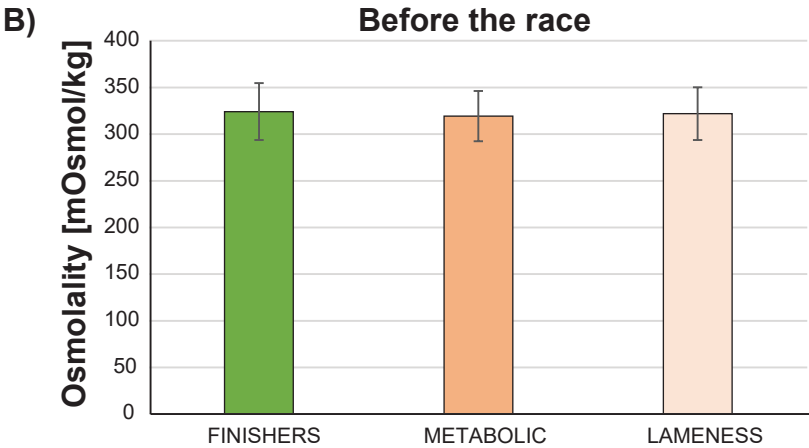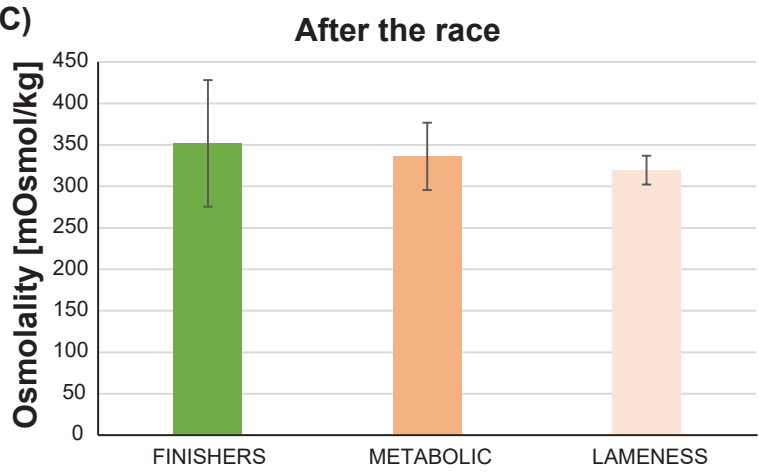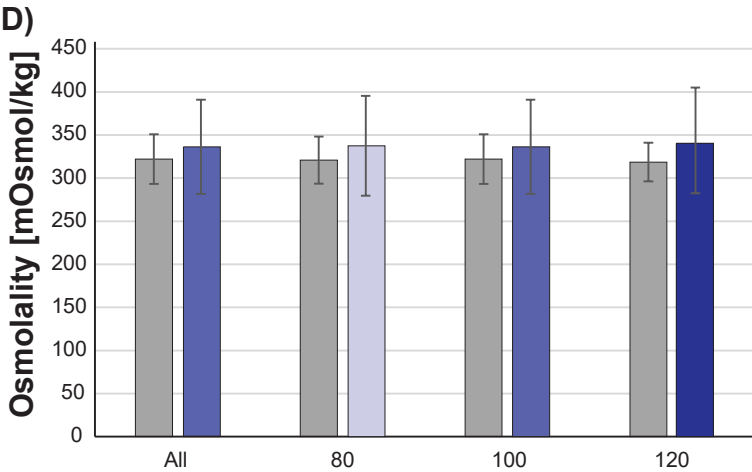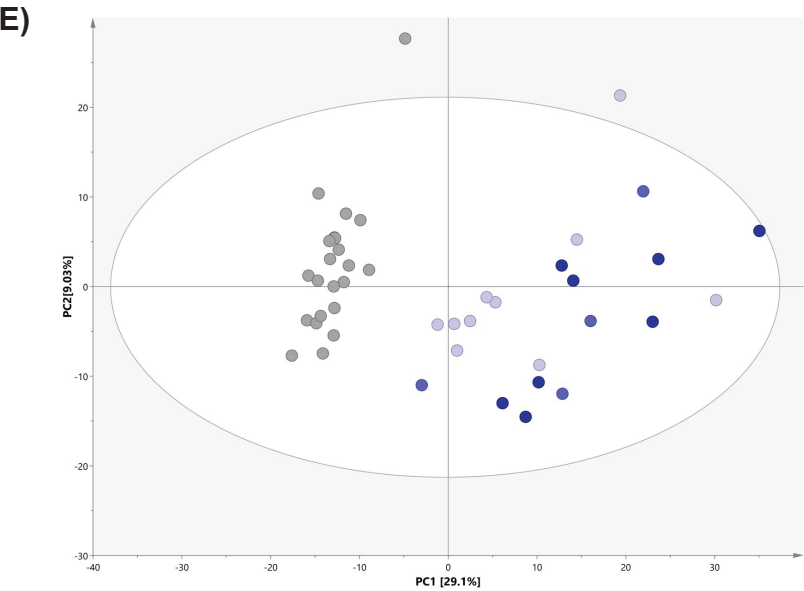

Supplement: Supplementary file 1 [file metabolites-11-00082-s001.zip › Supplementary Figure 1_V2.pdf]

FigS2

80 km

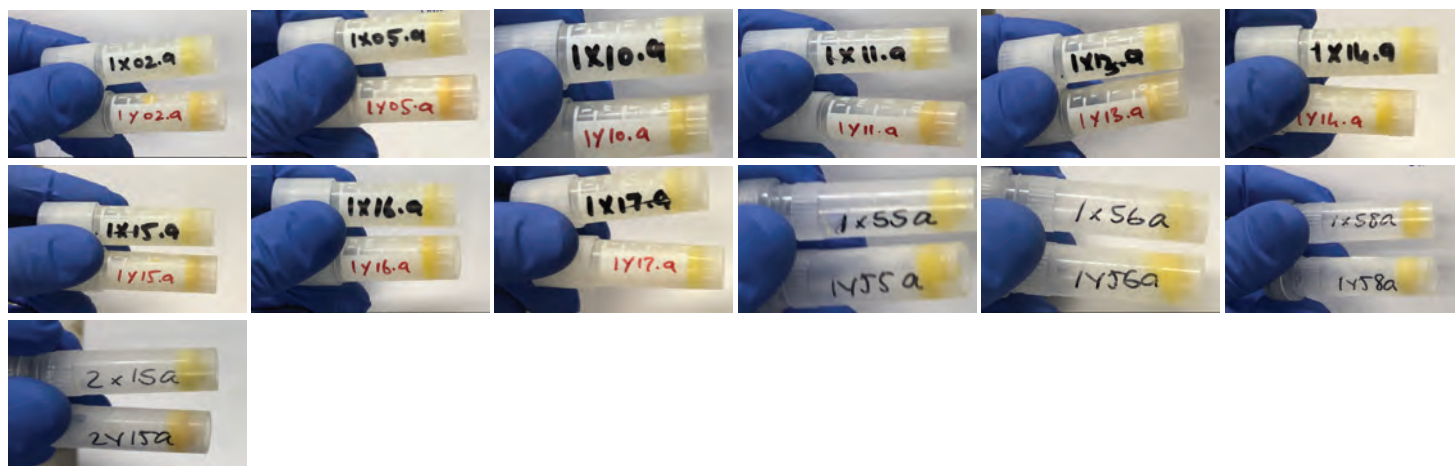

100 km

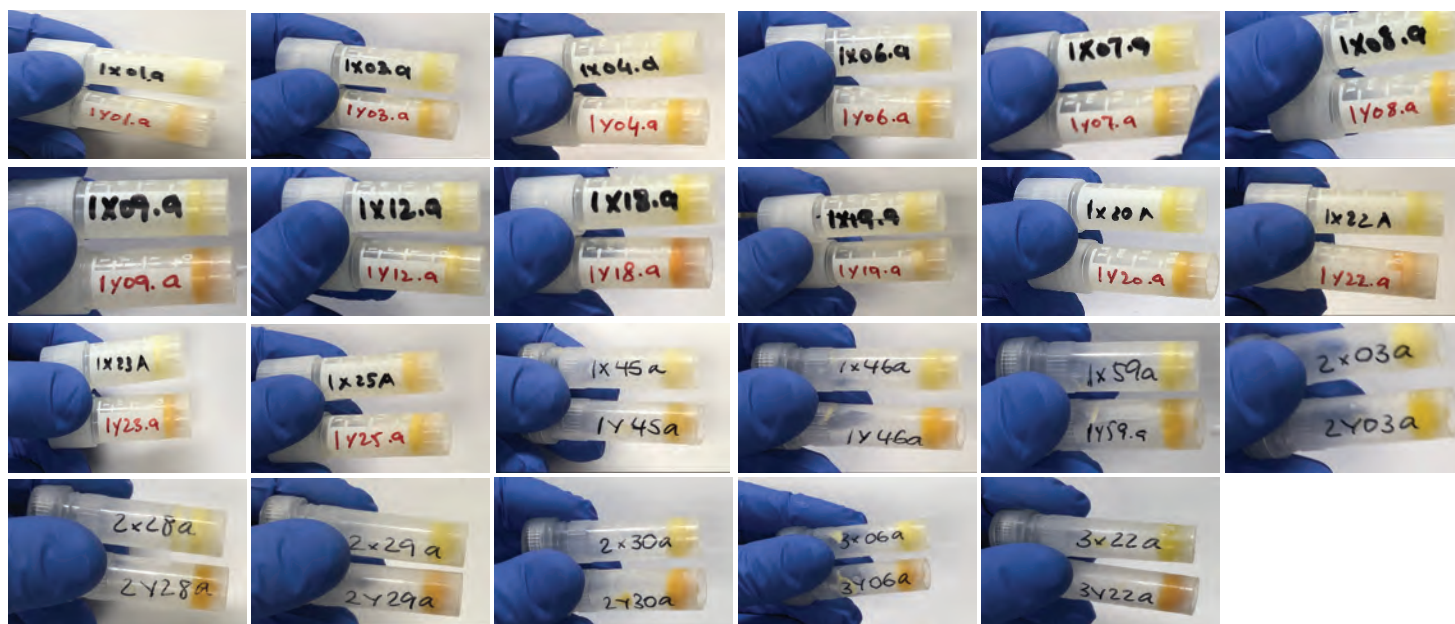

120 km

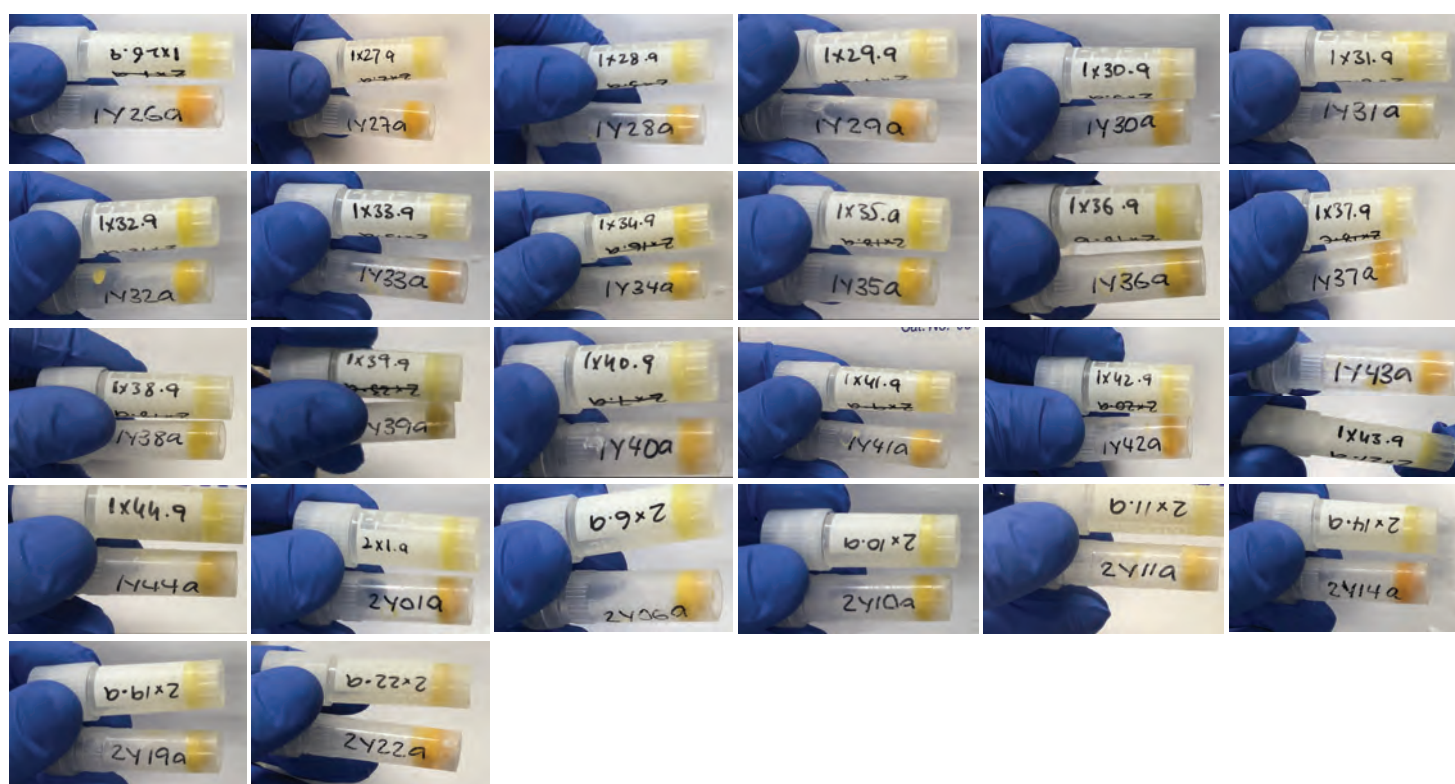

Supplement: Supplementary file 1 [file metabolites-11-00082-s001.zip › Supplementary Figure 2_Final.pdf]

FigS3

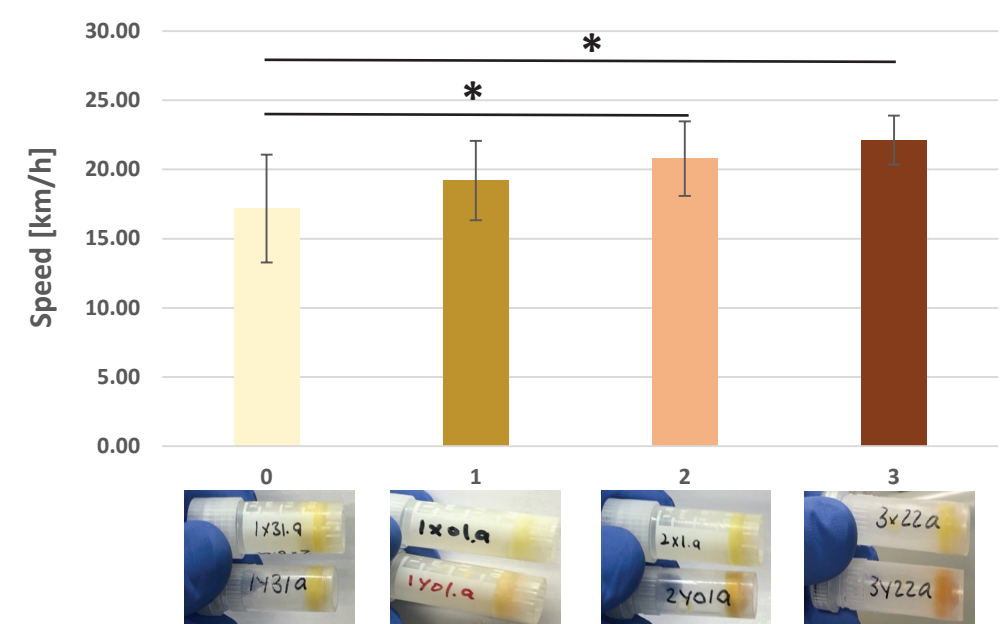

Supplement: Supplementary file 1 [file metabolites-11-00082-s001.zip › Supplementary Figure 3.pdf]
